# Performance evaluation of logarithmic spiral search and selective mechanism based arithmetic optimizer for parameter extraction of different photovoltaic cell models

**DOI:** 10.1371/journal.pone.0308110

**Published:** 2024-07-29

**Authors:** Erdal Eker, Davut Izci, Serdar Ekinci, Mohammad Shukri Salman, Mostafa Rashdan

**Affiliations:** 1 Vocational School of Social Sciences, Muş Alparslan University, Muş, Turkey; 2 Department of Computer Engineering, Batman University, Batman, Turkey; 3 Applied Science Research Center, Applied Science Private University, Amman, Jordan; 4 MEU Research Unit, Middle East University, Amman, Jordan; 5 College of Engineering and Technology, American University of the Middle East, Al Ahmadi, Kuwait; SRM-RI: SRM Institute of Science and Technology (Deemed to be University) Research Kattankulathur, INDIA

## Abstract

The imperative shift towards renewable energy sources, driven by environmental concerns and climate change, has cast a spotlight on solar energy as a clean, abundant, and cost-effective solution. To harness its potential, accurate modeling of photovoltaic (PV) systems is crucial. However, this relies on estimating elusive parameters concealed within PV models. This study addresses these challenges through innovative parameter estimation by introducing the logarithmic spiral search and selective mechanism-based arithmetic optimization algorithm (Ls-AOA). Ls-AOA is an improved version of the arithmetic optimization algorithm (AOA). It combines logarithmic search behavior and a selective mechanism to improve exploration capabilities. This makes it easier to obtain accurate parameter extraction. The RTC France solar cell is employed as a benchmark case study in order to ensure consistency and impartiality. A standardized experimental framework integrates Ls-AOA into the parameter tuning process for three PV models: single-diode, double-diode, and three-diode models. The choice of RTC France solar cell underscores its significance in the field, providing a robust evaluation platform for Ls-AOA. Statistical and convergence analyses enable rigorous assessment. Ls-AOA consistently attains low RMSE values, indicating accurate current-voltage characteristic estimation. Smooth convergence behavior reinforces its efficacy. Comparing Ls-AOA to other methods strengthens its superiority in optimizing solar PV model parameters, showing that it has the potential to improve the use of solar energy.

## Introduction

In recent years, the escalating environmental degradation and the dire consequences of climate change attributed to the excessive use of traditional fossil fuels like coal, oil, and gas have spurred a surge of interest in renewable energy sources [1]. Among these sustainable alternatives, solar energy shines as one of the most promising due to its clean, abundant, cost-effective, and ubiquitous nature [2]. Photovoltaic (PV) systems efficiently harness this boundless energy source, with precise modeling intimately linking the system’s performance accuracy [3–5].

PV system modeling widely employs three prominent PV cell models: the single-diode model, the double-diode model, and the more intricate three-diode model. However, these models harbor certain elusive physical parameters that remain undisclosed in PV manufacturer datasheets [6]. Accurate estimation of these hidden parameters is essential for a variety of aspects such as performance evaluation, quality control, and the critical task of maximum power point tracking in PV systems [7].

Understanding the parameters that characterize the behavior of solar cells and modules is of paramount importance for their efficient utilization and integration into renewable energy systems. In this regard, parameter estimation techniques have emerged as crucial tools. For example, Jiang et al. [8] introduced an improved adaptive differential evolution algorithm for the parameter estimation of solar cells and modules. The algorithm demonstrated its effectiveness in optimizing solar PV models, contributing to the accurate characterization of these energy conversion systems. Oliva et al. [9] proposed the use of an improved chaotic whale optimization algorithm for parameter estimation of photovoltaic cells. This approach harnesses chaotic dynamics to enhance the exploration-exploitation balance of the optimization process. The algorithm exhibited notable accuracy in parameter estimation, contributing to the advancement of solar cell modeling. Long et al. [10] presented a novel hybrid algorithm that combines the grey wolf optimizer and cuckoo search for parameter extraction in solar PV models. This approach achieved precise parameter estimation by combining the strengths of these two optimization techniques. The study showcased the efficacy of hybrid algorithms in enhancing parameter estimation accuracy. Jiao et al. [11] introduced the orthogonally adapted Harris hawks optimization algorithm for parameter estimation of photovoltaic models. Inspired by the hunting behavior of hawks, this algorithm demonstrated an orthogonal adaptation strategy, improving parameter estimation accuracy. This innovative approach offered a unique perspective on optimizing solar PV models. Abdelghany et al. [12] developed an improved bonobo optimizer and applied it to solar cell parameter estimation. This study showcased the algorithm’s capabilities in accurately characterizing solar cells. The improved Bonobo optimizer introduced innovations that contributed to parameter estimation precision, further enhancing our understanding of solar energy systems. Houssein et al. [13] introduced the manta ray foraging optimization algorithm for parameter extraction in three-diode photovoltaic models. Inspired by the foraging behavior of manta rays, this algorithm demonstrated efficiency in parameter estimation. It provided a unique perspective on optimizing complex solar PV models, particularly the three-diode model. Ayyarao and Kumar [14] presented a novel war strategy optimization algorithm for parameter estimation of solar PV models. This innovative approach drew inspiration from war strategies to optimize complex systems. The algorithm exhibited notable accuracy in characterizing solar PV models, providing a fresh perspective on parameter estimation techniques.

As discussed above, numerous methodologies and algorithms have emerged to tackle the challenge of parameter estimation in PV models. These algorithms have demonstrated their effectiveness in accurately characterizing solar cells and modules, contributing to the efficient utilization of solar energy. Each approach brings unique strengths and innovations, paving the way for further developments in the field of renewable energy [NO_PRINTED_FORM]. Yet, several research gaps persist [15]. Firstly, many metaheuristic algorithms come laden with control parameters that demand meticulous tuning for optimal performance in each problem domain [16]. In response to this challenge, some parameter-free metaheuristic methods have emerged [17,18]. However, these approaches exhibit drawbacks such as sluggish convergence and inadequate population diversity [19]. Second, because of the inherent stochasticity of metaheuristics, their convergence rates and stability occasionally prove unsatisfactory. To surmount these issues, hybrid algorithms [20–23] have been introduced, combining metaheuristics with deterministic local search techniques. Although these hybrids offer improved performance, they often rely on gradient information and necessitate laborious parameter tuning processes. Lastly, while existing research predominantly concentrates on estimating parameters for single-diode and double-diode models, the three-diode model, owing to its complexity, offers a superior means of evaluating algorithmic performance. Regrettably, only a limited number of approaches have ventured into the realm of the three-diode model [24,25].

This paper embarks on the critical task of parameter estimation in PV models, with a keen focus on enhancing existing methodologies. Motivated by the identified gaps, we introduce the logarithmic spiral search and selective mechanism-based arithmetic optimization algorithm (Ls-AOA) [26] as an innovative and potent metaheuristic tool for parameter estimation in PV models. Ls-AOA represents an improved iteration of the original arithmetic optimization algorithm (AOA) [27], incorporating the synergy of logarithmic search behavior and a selective mechanism. By harnessing these logarithmic search and selective mechanisms, the algorithm enhances its exploration capabilities, rendering it a valuable asset for extracting precise parameters in PV models.

This study focuses on the RTC France solar cell as a case study, with the goal of maintaining consistency and impartiality. To ensure uniformity, we established a standardized experimental framework. The incorporation of the Ls-AOA introduced a systematic and efficient approach to navigate the intricate parameter space of solar PV models. We integrated logarithmic spiral search and selective mechanisms into the AOA framework in order to fine-tune the parameters of three different models: the single-diode model (SDM), the double-diode model (DDM), and the three-diode model (TDM). We chose the RTC France solar cell as benchmark case study due to its significant role in the solar photovoltaics field, which serves as a robust testbed for evaluating the effectiveness of Ls-AOA across various solar cell models.

In our pursuit of rigorous assessment and interpretation of findings, we executed statistical and convergence analyses. These analyses provided invaluable insights, empowering us to draw substantial conclusions regarding the Ls-AOA’s efficacy in optimizing diverse solar cell models. The experimental results of the SDM, DDM and TDM optimizations using the Ls-AOA show high accuracy in parameter estimation. The proposed algorithm consistently achieves low RMSE values, indicating its superior performance in accurately estimating the current-voltage (I-V) characteristics. The Ls-AOA’s convergence behavior demonstrates smooth convergence. The performance metrics of the Ls-AOA show close agreement between experimental and estimated values, demonstrating the accurate modeling capabilities of the proposed approach. We also delve into a comprehensive statistical analysis comparing the Ls-AOA’s performance with alternative methods, including hybrid multi-group stochastic cooperative particle swarm optimization [28], improved learning search algorithm [29], generalized oppositional teaching learning based optimization [30], teaching—learning—based artificial bee colony [31], inspired grey wolf optimizer [32], improved opposition-based whale optimization algorithm [33], sunflower optimization algorithm [34], gradient-based optimizer [35], spherical evolution [36], slime mould algorithm [37], atom search optimization [38], comprehensive learning particle swarm optimizer [39], particle swarm optimization [40], hybrid rat swarm optimization and pattern search [41], modified salp swarm optimization [42], hybrid single candidate optimizer and chaotic sand cat optimizer [43] and improved tunicate swarm optimization [44], further establishing its competitive edge in the realm of solar PV model parameter optimization. Considering the above discussion, the key contributions of this work can be listed as follows.

- Introduction of the logarithmic spiral search and selective mechanism-based arithmetic optimization algorithm (Ls-AOA) for parameter estimation in PV models.
- Demonstration of the effectiveness of the Ls-AOA in optimizing diverse solar cell models, including the single-diode model, double-diode model, and three-diode model.
- Proposal of a novel approach integrating the Ls-AOA with the Newton-Raphson method for the complete inference of solar PV system parameters.
- Evaluation of the proposed approach on three different diode models, along with rigorous statistical analysis and RMSE convergence curve performances of the Ls-AOA algorithm on these models.
- Comparison with popular and contemporary methods in the literature, showcasing its competitive edge in solar PV model parameter optimization.

## Background of the original AOA

The AOA draws inspiration from arithmetic principles, as proposed by Abualigah et al. [27]. A collection of random solutions is produced at the beginning of the process, as demonstrated by Eq (1), during the initialization stage.


$$X=\left[\begin{array}{cccccc} x_{1,1} & \cdots & \cdots & x_{1,j} & x_{1,n-1} & x_{1,n}\\ x_{2,1} & \cdots & \cdots & x_{2,j} & \cdots & x_{2,n}\\ \cdots & \cdots & \cdots & \cdots & \cdots & \cdots \\ \vdots & \vdots & \vdots & \vdots & \vdots & \vdots \\ x_{N-1,1} & \cdots & \cdots & x_{N-1,j} & \cdots & x_{N-1,n}\\ x_{N,1} & \cdots & \cdots & x_{N,j} & x_{N,n-1} & x_{N,n} \end{array}\right]$$ (1)

Following the initialization stage, the algorithm employs a function known as "Math Optimizer Accelerated" (*MopA*) described in Eq (2) to perform explorative and exploitative tasks:

$$MopA\left(t\right)=Min+t\times \left(\frac{Max-Min}{t_{Max}}\right)$$ (2)

where *t* represents the current iteration and *t*_*Max*_ is the maximum number of iterations. *MopA*(*t*) represents the value of the function at the current iteration. *Min* and *Max* denote the minimum and maximum values of the accelerated function.

The explorative phase of the AOA occurs when *r*_1_ > *MopA* where *r*_1_ is a random number. The exploration process involves multiplication (*Mult*) and division (*Div*) operations, as explained in Eq (3). Here, *x*_*i*_(*t +* 1*)* represents the solution *i* in the next iteration, *x*_*i*,*j*_(*t*) represents the *j*^*th*^ position of solution *i* in the current iteration, and *best*(*x*_j_) represents the *j*^*th*^ position of the best solution obtained so far.


xi,jt+1=bestxj×MopP×UBj-LBj×μ+LBj,forr2>0.5bestxj÷MopP+ϵ×UBj-LBj×μ+LBj,forr2<0.5 (3)

Here, *ϵ* is a small integer, and *μ* is a control parameter used to adjust the search process. *UB*_*j*_ and *LB*_*j*_ represent the upper and lower bounds of the *j*^*th*^ position, respectively. *MopP* is a function called "Math Optimizer Probability" calculated according to Eq (4):

$$MopP\left(t\right)=1-\frac{{\left(t\right)}^{1/\alpha }}{{\left(t_{Max}\right)}^{1/\alpha }}$$ (4)

where *α* denotes the exploitation accuracy through iterations. A random number *r*_2_ is used to decide whether to perform the multiplication (*Mult*) or division (*Div*) operation in Eq (3), with *Mult* occurring for *r*_2_ > 0.5 and Div *r*_2_ ≤ 0.5. Conversely, the exploitative phase is executed when *r*_1_ < *MopA*, employing addition (*Add*) and subtraction (*Sub*) operators as modeled in Eq (5):

xi,jt+1=bestxj+MopP×UBj-LBj×μ+LBj,forr3>0.5bestxj-MopP×UBj-LBj×μ+LBj,forr3<0.5 (5)

where *r*_3_ is a random number determining which operator to employ. In summary, the AOA combines exploration and exploitation phases guided by random numbers and mathematical optimization functions, leveraging various operators to update solution values during the search process.

## Logarithmic spiral search and selective mechanisms-based AOA

**Fig 1:**
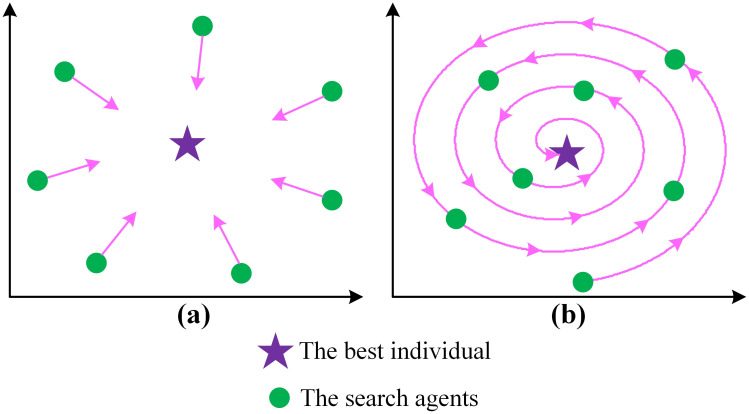
The original (a) and the logarithmic spiral (b) search models.

It is possible to improve the original AOA by incorporating more advanced exploratory features, achieving a balanced combination of exploration and exploitation stages [45,46]. Fig 1(a) demonstrates that the search agents in the original AOA have a tendency to progressively approach the optimal solution in a linear manner with each iteration. This indicates a strong potential to intensify, but it comes at the cost of having a limited diversity of population. As a result, it is prone to get stuck in local optima. Therefore, this work utilizes a logarithmic spiral search technique, illustrated in Fig 1(b), with the purpose of providing the original AOA with the intended population variety. The mathematical expression for this model is given by Eq (6), where *l* is a random variable within the range [−1,1], calculated as *l* = 2 × *rand* −1, *α* is a constant set to 1, shaping the spiral, and *x*_*best*_(*t*) represents the optimal position in the current iteration.


$$x_{i}^{Ls}\left(t\right)=\left|x_{best}\left(t\right)-x_{i}\left(t\right)\right|\cdot e^{\alpha l}\cdot \text{cos}\;\left(2\pi l\right)+x_{best}\left(t\right)$$ (6)

As depicted in Fig 1(b), individuals update their positions during each iteration, converging toward the target in a spiral fashion, thereby preserving the population’s diversity. This approach amplifies the algorithm’s exploration capabilities. Moreover, the proposed algorithm incorporates a selective structure to further enhance its performance. Consequently, the position update mechanism is adapted using the following definition, where *x*_*i*_(*t* + 1) represents the *i*^*th*^ search agent in the subsequent iteration.


xit+1=xiLst,fitnessxiLst≤fitnessxitxit,fitnessxiLst>fitnessxit (7)

**Fig 2:**
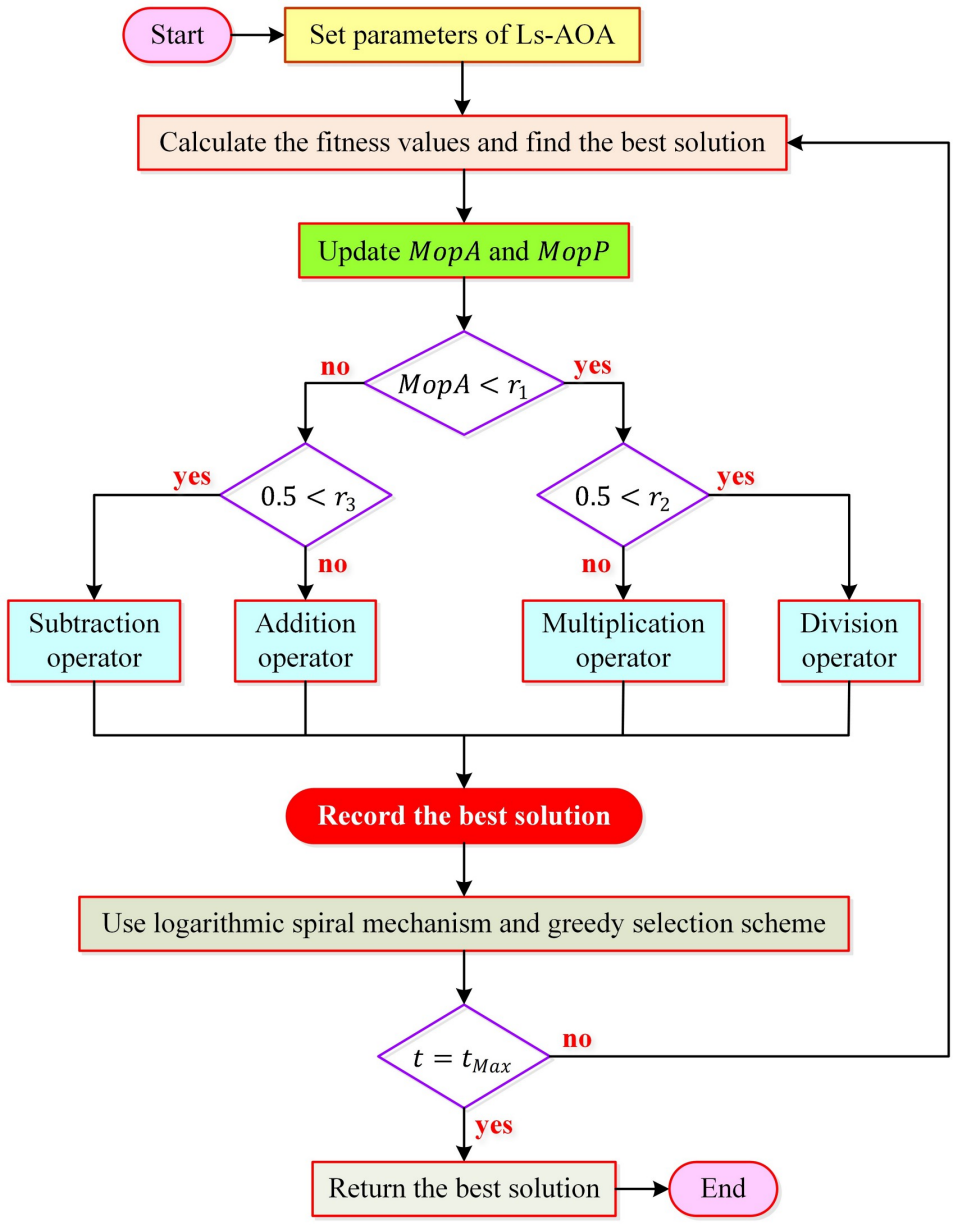
Flowchart for recommended Ls-AOA optimizer.

According to Eq (7), the current solution, *x*_*i*_(*t*), is replaced by the newly obtained solution, $x_{i}^{Ls}\left(t\right)$, in the event that *x*_*i*_(*t*) exhibits equal or superior fitness. Otherwise, *x*_*i*_(*t*) remains within the population. This selection mechanism effectively prevents the retention of suboptimal solutions. In essence, superior new solutions are continually refined over successive iterations, while inferior ones are systematically discarded until the termination criterion (*t* = *t*_*Max*_) is met. A comprehensive flowchart outlining the proposed logarithmic spiral search and selective mechanism-based AOA (Ls-AOA) is provided in Fig 2. This Ls-AOA has previously been reported in one of our previous works [26]. In this study, we further investigate its performance for another challenging real-world engineering problem, namely parameter extraction of photovoltaic system through different cell models.

## Problem formulation of solar PV system

### Single Diode Model (SDM)

**Fig 3:**
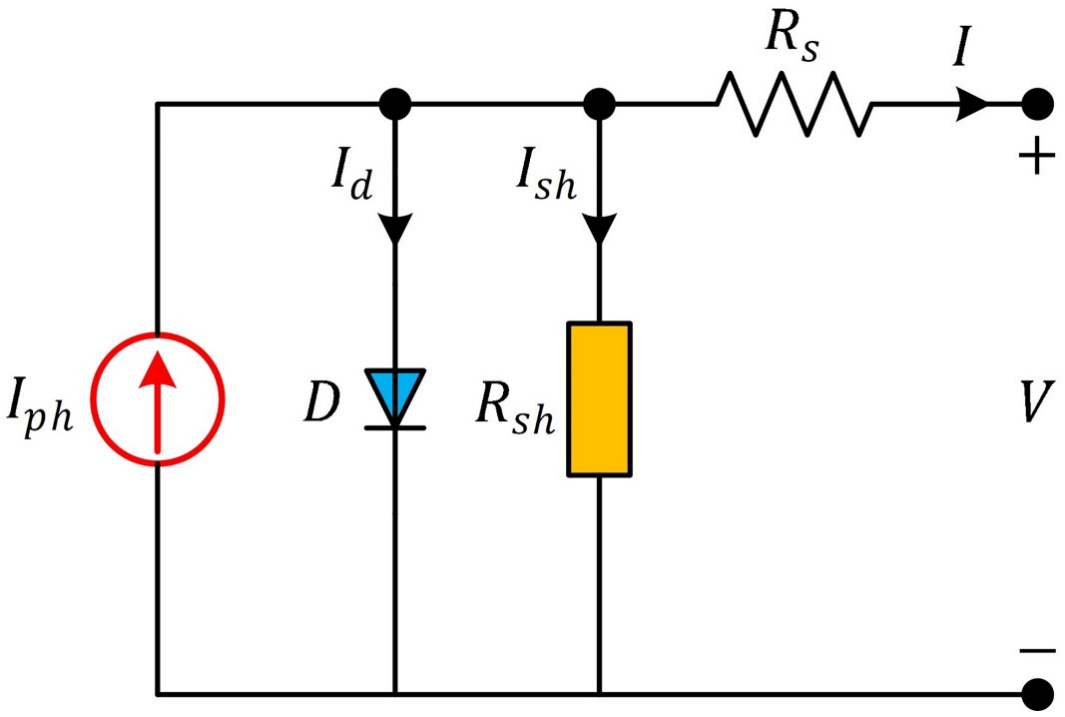
Equivalent circuit of SDM.

The SDM offers a simplified mathematical representation of the electrical characteristics exhibited by a PV cell. It assumes that the PV cell can be effectively modeled as a single diode connected in parallel with a current source. Despite its simplicity, the SDM manages to capture the essential aspects of the PV cell’s electrical response while providing a computationally efficient representation. In the SDM, the I-V relationship of a PV cell is defined by the following equation:

$$I=I_{ph}-I_{sd}[e^{\frac{\left(V+IR_{s}\right)}{\left(nV_{t}\right)}}-1]-\frac{\left(V+IR_{s}\right)}{R_{sh}}$$ (8)

where *I* is the output current of the PV cell, *V* is the voltage across the PV cell terminals, *I*_*ph*_ is the photocurrent generated by the cell under illumination, *I*_*sd*_ is the diode saturation current, *R*_*s*_ is the series resistance of the cell, *R*_*sh*_ is the shunt resistance of the cell, *n* is the diode ideality factor, *V*_*t*_ is the thermal voltage, approximately equal to *kT*/*q*, where *k* is Boltzmann’s constant, *T* is the temperature in Kelvin, and *q* is the elementary charge. Fig 3 illustrates the conceptual depiction of a solar PV cell employing the single-diode model.

### Double Diode Model (DDM)

**Fig 4:**
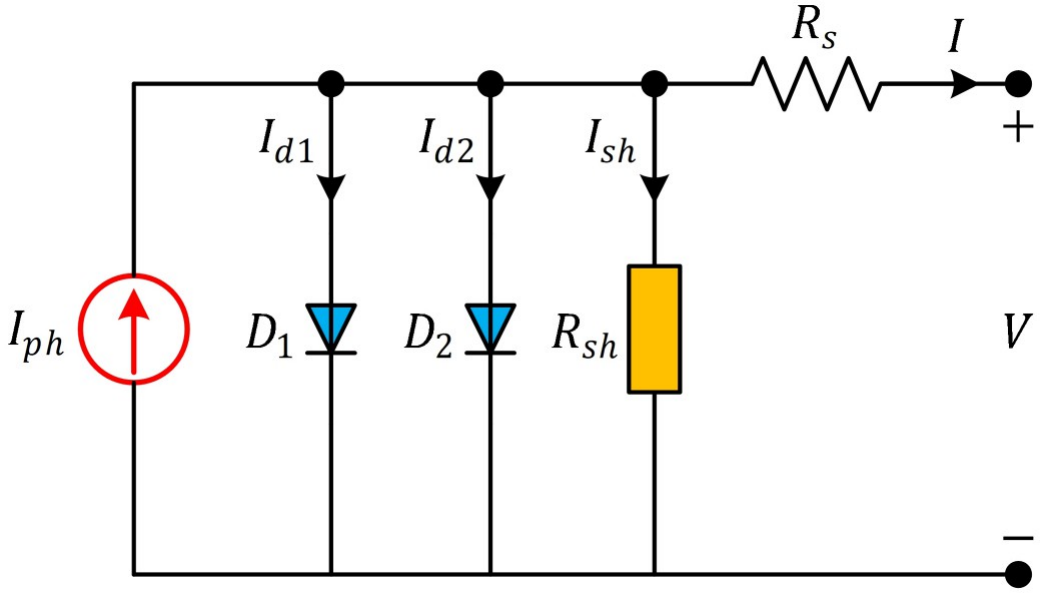
Equivalent circuit of DDM.

The DDM represents an advanced approach to PV cell modeling that incorporates additional diodes to capture more complex electrical behavior. By introducing an extra diode to account for recombination losses within the PV cell, the DDM offers a more precise description of real-world PV cell characteristics. In the DDM, the I-V relationship of a PV cell is defined by the following equation:

$$I=I_{ph}-I_{sd1}[e^{\frac{\left(V+IR_{s}\right)}{\left(n_{1}V_{t}\right)}}-1]-I_{sd2}[e^{\frac{\left(V+IR_{s}\right)}{\left(n_{2}V_{t}\right)}}-1]-\frac{\left(V+IR_{s}\right)}{R_{sh}}$$ (9)

where *I*_*sd*1_ is the diode saturation current of the main diode, *I*_*sd*2_ is the diode saturation current of the additional diode, *n*_1_ is the ideality factor of the main diode and *n*_2_ is the ideality factor of the additional diode. Fig 4 illustrates the conceptual depiction of a solar PV cell employing the double-diode model. Compared to the SDM, the DDM offers a more accurate representation of the electrical characteristics of PV cells, particularly in situations where recombination losses significantly impact performance. However, due to the increased complexity of the model, parameter estimation for the DDM can be more computationally intensive and requires a larger number of measurement data points to achieve reliable results.

### Three Diode Model (TDM)

**Fig 5:**
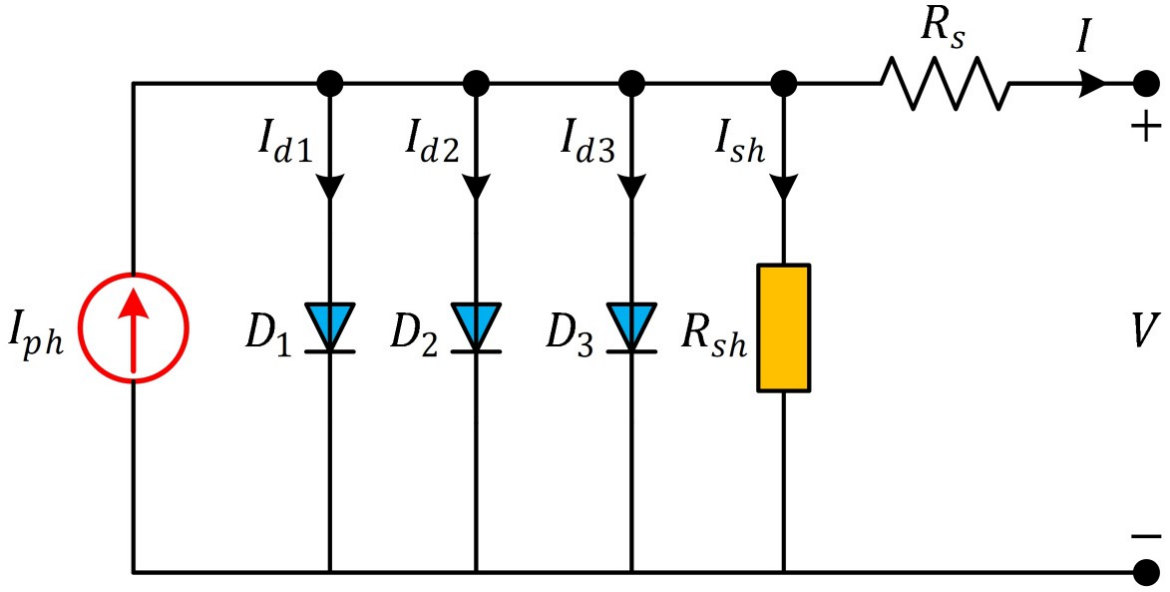
Equivalent circuit of TDM.

The TDM is an advanced representation of a PV cell that provides a more accurate description of its behavior compared to simpler models. It takes into account various factors that affect the cell’s performance, such as recombination, shunt resistance, and series resistance. The model divides the cell into three diodes: the ideal diode, the recombination diode, and the shunt diode. The ideal diode represents the basic behavior of a PV cell when it’s illuminated and generating power. It accounts for the conversion of light energy into electrical current, considering the cell’s short-circuit current and open-circuit voltage. The current-voltage relationship for the ideal diode is given by the Shockley diode equation:

$$I=I_{ph}-I_{sd1}-I_{sd2}-I_{sd3}-I_{sh}$$ (10)

where *I*_*sd*1_ is the current through the ideal diode; *I*_*sd*2_ is the current through the recombination diode and *I*_*sd*3_ is the current through the shunt diode. Considering this explanation, the overall current through the TDM can be calculated by summing up the currents through the three diodes in the model:

$$I=I_{ph}-I_{sd1}\left(e^{\frac{V+IR_{s}}{n_{1}V_{t}}-1}\right)-I_{sd2}\left(e^{\frac{V+IR_{s}}{n_{n2}V_{t}}-1}\right)-I_{sd3}\left(e^{\frac{V+IR_{s}}{n_{3}V_{t}}-1}\right)-\frac{V+IR_{s}}{R_{sh}}$$ (11)

where *n*_*d*1_, *n*_*d*2_ and *n*_*d*3_ are the ideality factors of the diodes *D*_1_, *D*_2_ and *D*_3_, respectively. Fig 5 illustrates the equivalent circuit of a solar PV cell employing the three-diode model. This three-diode model provides a more accurate representation of the behavior of a photovoltaic cell under different operating conditions, accounting for recombination losses and shunt resistance, which are important factors affecting the cell’s efficiency and performance.

### Objective function and application of Newton-raphson aided Ls-AOA

In the process of estimating parameters for photovoltaic (PV) cells, the objective function plays a pivotal role in evaluating the accuracy of the estimated parameters. It achieves this by comparing the modeled I-V curve with the actual measured data. Among the various metrics available for assessing the goodness of fit, root mean square error (RMSE) stands out as a commonly employed choice. RMSE, a widely accepted measure, quantifies the average magnitude of differences between the estimated current (*I*_*est*_) and the experimentally measured current (*I*_*m*_). The formula for RMSE is given by Eq (12):

$$RMSE=\sqrt{\frac{1}{N}\sum_{i=1}^{N}{\left(I_{m}-I_{est}\right)}^{2}}$$ (12)

Here, *N* represents the total number of data points. RMSE offers a comprehensive assessment of the overall disparity between the model and the measured data. By squaring the differences, RMSE assigns greater weight to larger errors, which proves beneficial when outliers or extreme values significantly influence the analysis. A lower RMSE value indicates a more favorable agreement between the model and the actual measurements.

**Fig 6:**
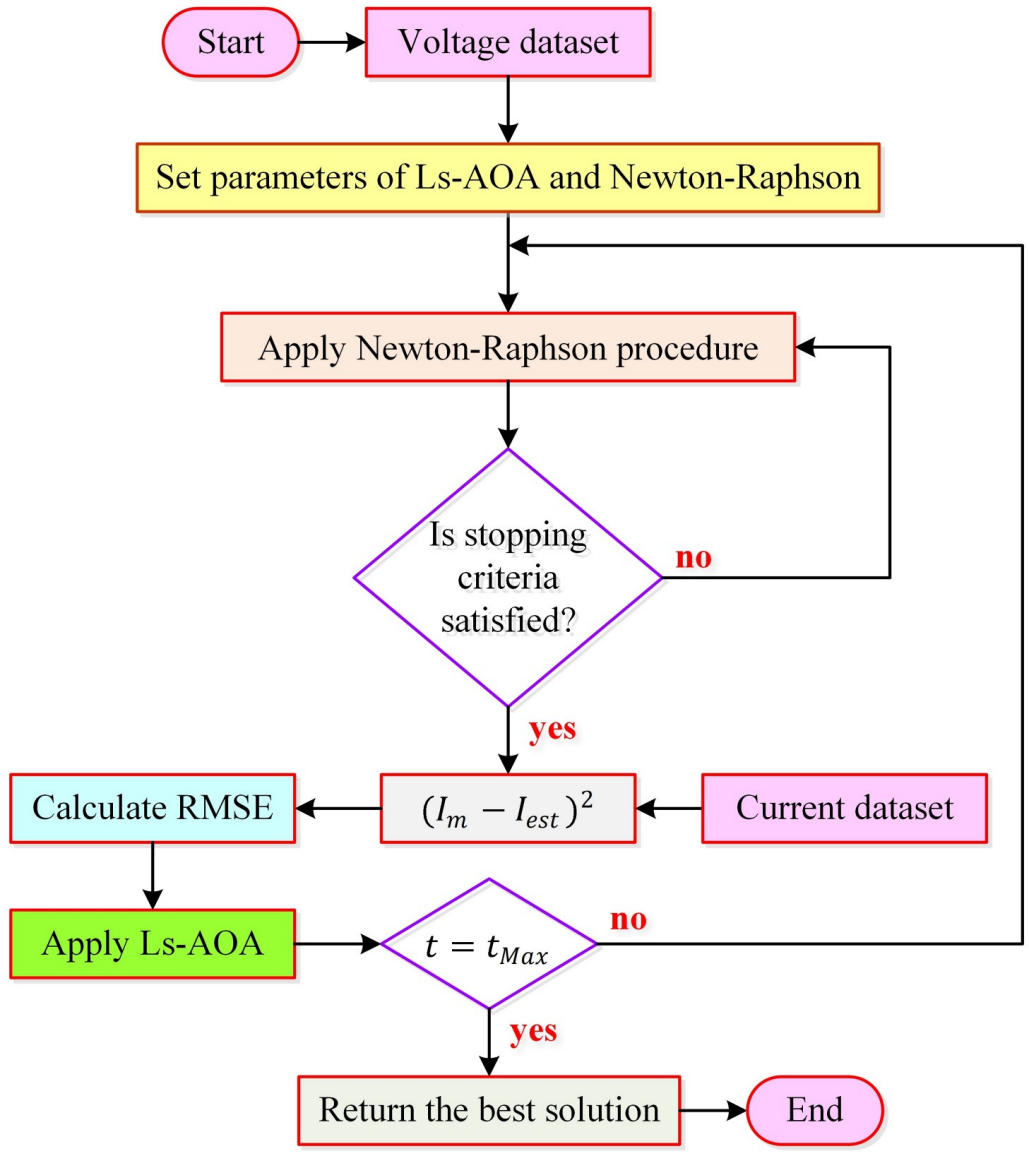
Parameter extraction process with the combination of Newton-Raphson method and Ls-AOA.

In the literature, various approaches including analytical, numerical, and metaheuristic methods have been employed for accurate parameter estimation of PV cells [47]. While analytical methods offer simplicity and speed, they often rely on numerous mathematical equations, some of which are derived based on simplifying assumptions, potentially limiting their accuracy [48]. To overcome this limitation, researchers have suggested techniques such as Taylor series expansion, Lambert W function, and Newton-Raphson [49]. Numerical methods, on the other hand, offer advantages in terms of reliability, robustness, simplicity, and ease of implementation. However, their performance can be significantly influenced by factors such as the selection of initial values and the number of variables to be estimated [48]. In our manuscript, we have carefully considered these factors and we have opted to employ the iterative Newton-Raphson method [48] for parameter extraction. This method boasts several advantages, including a relatively low computational burden and high accuracy. The algorithm is seamlessly integrated with the Newton-Raphson method, ensuring their synergy throughout the parameter estimation process. Fig 6 visually illustrates the parameter extraction process by combining the Newton-Raphson method with the proposed Ls-AOA.

## Simulation results and discussion

In this section, the experimental findings and ensuing statistical analyses are presented, with the Ls-AOA being utilized to determine the parameters of solar PV models. The examination focuses on the RTC France solar cell as a case study. To ensure consistency and impartiality, a fixed population size of 30 and a maximum iteration limit of 400 were set for all experiments. Additionally, each case study underwent 25 iterations to accommodate potential variations in the optimization process.

The adoption of the Ls-AOA facilitated a systematic and efficient exploration of the parameter space of solar PV models. Through the seamless integration of logarithmic spiral search and selective mechanisms into the AOA framework, the parameters were fine-tuned to optimize the performance of three different models: SDM, DDM, and TDM. The selection of the RTC France solar cell as the benchmark case study was driven by its significance in the field of solar photovoltaics. Utilization of SDM, DDM, and TDM allowed assessment of the effectiveness of the Ls-AOA across various solar cell models. Detailed parameter limits for SDM, DDM, and TDM are provided in Table 1.

**Table 1:** The parameter bounds of SDM, DDM and TDM.

Parameter	Minimum	Maximum
*I*_*ph*_ (A)	0	1
*I*_*sd*_ (μA)	0	1
*I*_*sd*1_, *I*_*sd*2_, *I*_*sd*3_ (μA)	0	1
*R*_*s*_ (Ω)	0	0.5
*R*_*sh*_ (Ω)	0	100
*n*	1	2
*n*_1_, *n*_2_, *n*_3_	1	2

To rigorously assess and interpret the findings, statistical and convergence analyses were conducted. These analyses yielded valuable insights, enabling meaningful conclusions to be drawn about the efficacy of the Ls-AOA in optimizing different solar cell models. Subsequently, the experimental setup is outlined in detail, the obtained results are discussed, and an extensive statistical analysis contrasting the performance of the Ls-AOA with alternative methods is presented.

### Simulation results of SDM

In this section, the experimental outcomes of the SDM optimization accomplished through the utilization of the proposed Ls-AOA are presented. Table 2 summarizes the estimated parameters of the SDM obtained via the Ls-AOA, shedding light on the algorithm’s capability to achieve remarkable accuracy in parameter estimation. This highlights the significant performance of the Ls-AOA in fine-tuning the SDM parameters.

**Table 2:** Estimated parameters of SDM with Ls-AOA.

*I*_*ph*_ (A)	*I*_*sd*_ (μA)	*R*_*s*_ (Ω)	*R*_*sh*_ (Ω)	*n*	RMSE
0.7608	0.3107	0.0365	52.8899	1.4773	7.7299E−04

**Fig 7:**
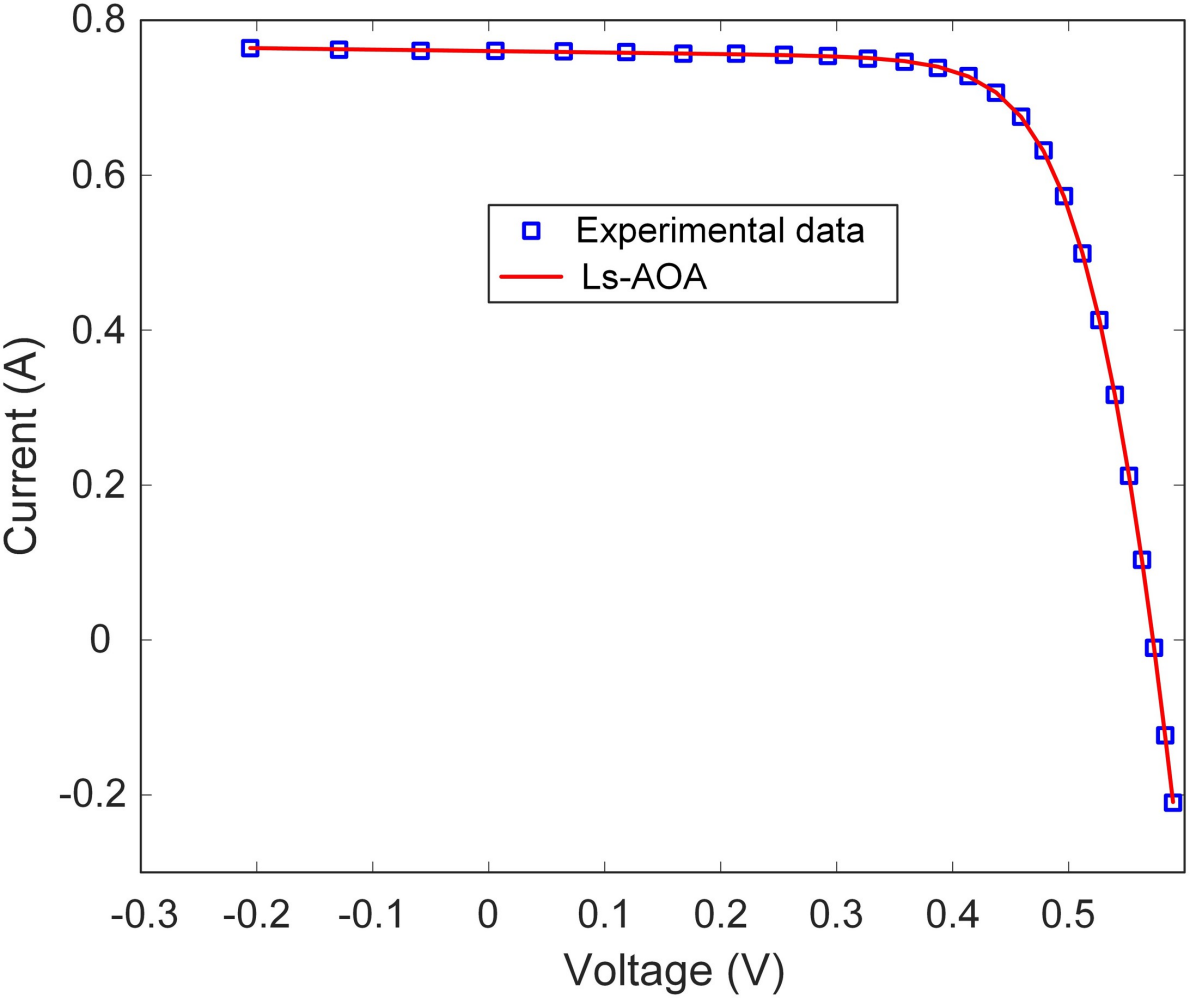
I-V curve characteristics of SDM.

**Fig 8:**
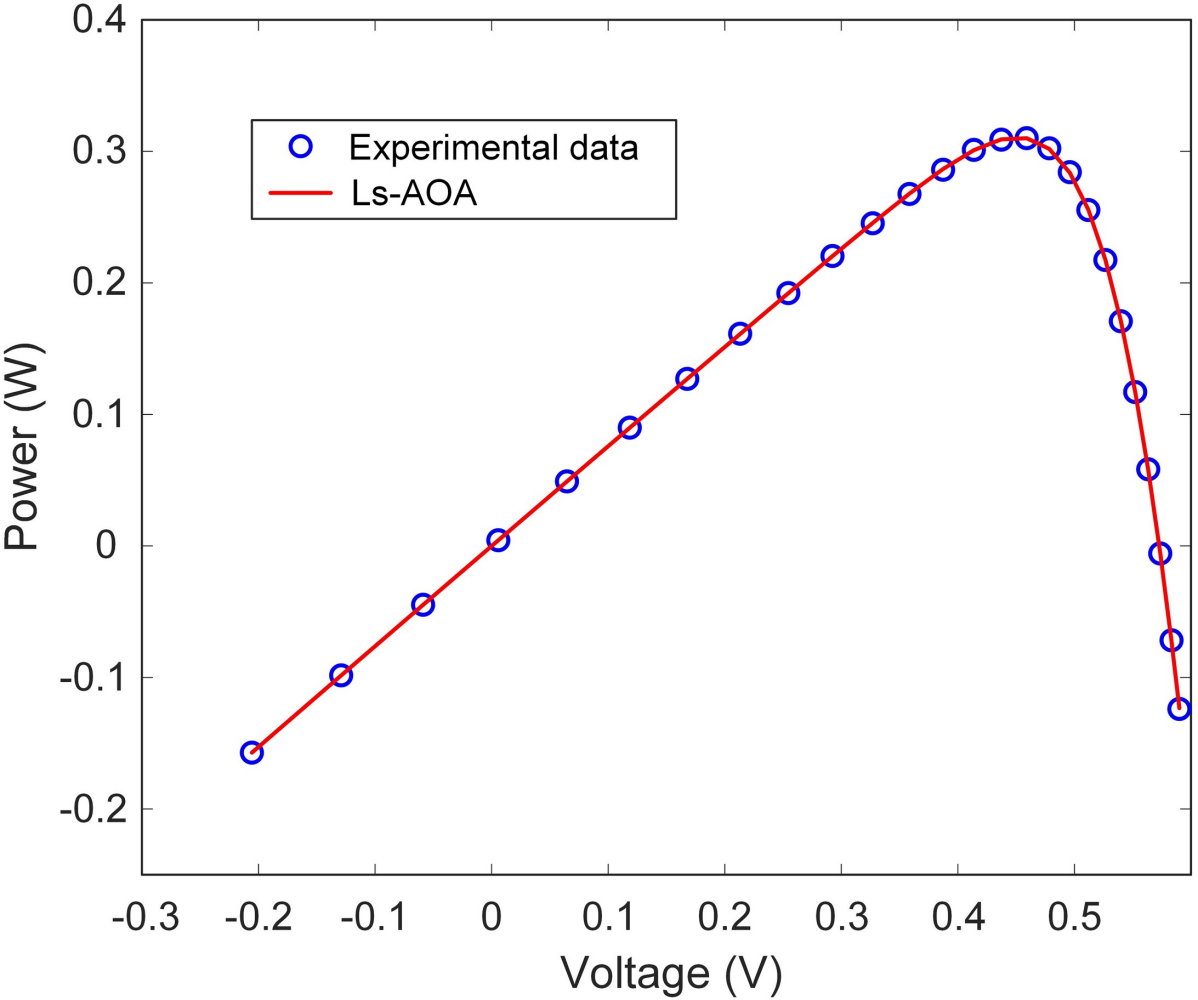
P-V curve characteristics of SDM.

Additionally, Table 2 reports the best RMSE value for the SDM. The results underscore the Ls-AOA’s ability to yield a low RMSE value, signifying precise estimation of the I-V characteristics of the SDM. Furthermore, as illustrated in Figs 7 and 8, we present the I-V and power-voltage (P-V) curve characteristics of the SDM optimized using the Ls-AOA. These figures effectively demonstrate that the optimized model adeptly captures the behavior of the solar cell, as evidenced by the close alignment of the curves with the experimental data.

### Simulation results of DDM

**Fig 9:**
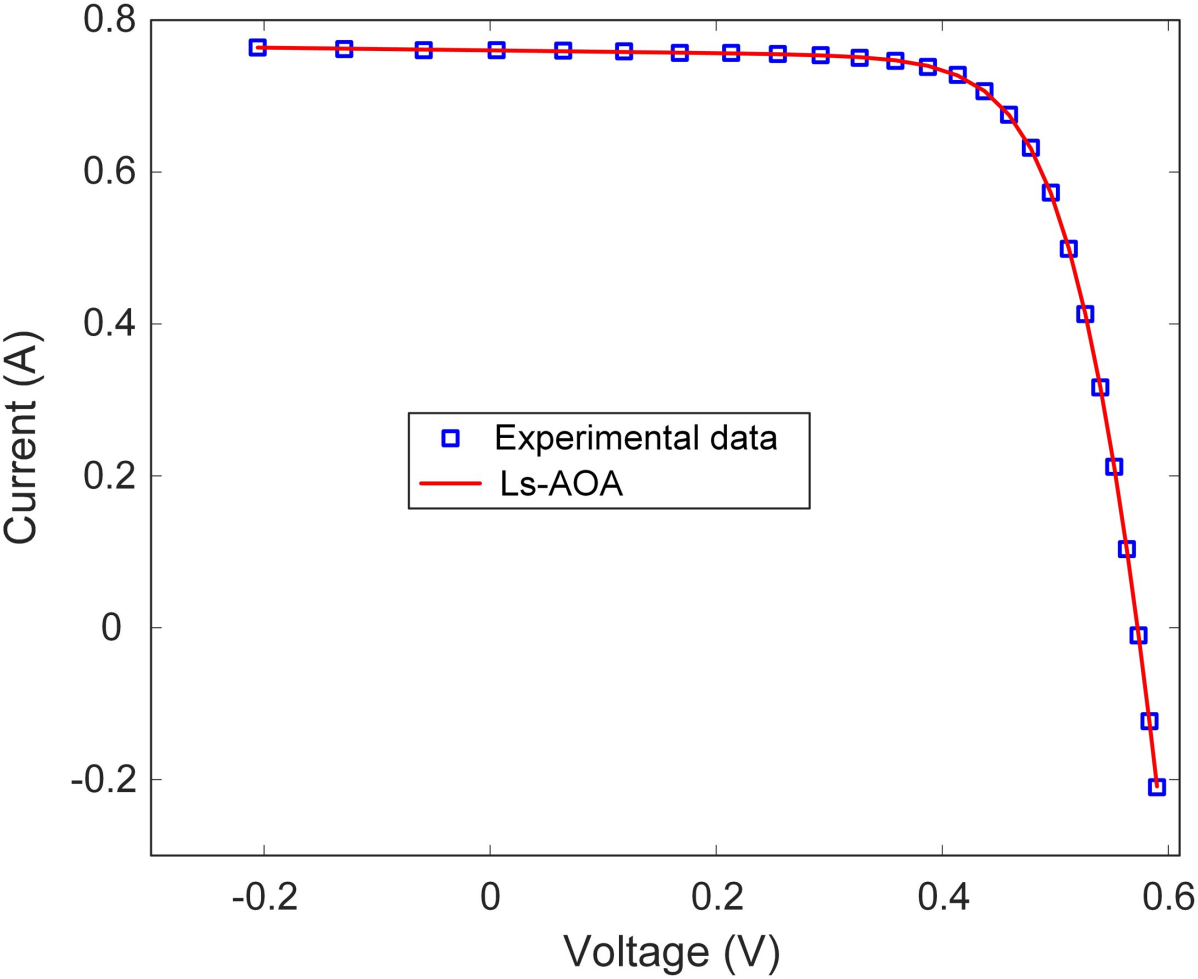
I-V curve characteristics of DDM.

**Fig 10:**
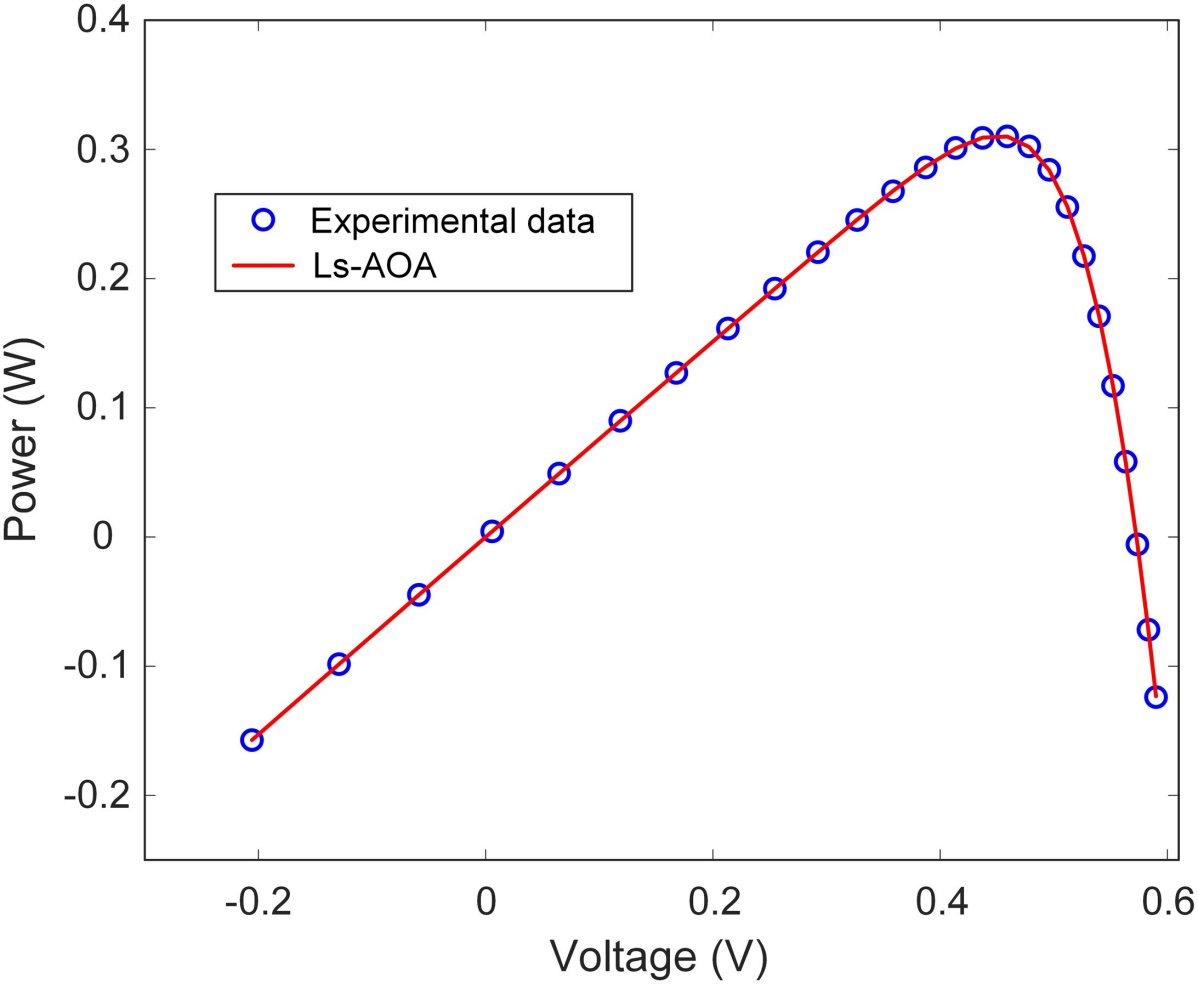
P-V curve characteristics of DDM.

In this section, the simulation results for the DDM optimization conducted using the Ls-AOA are presented. Table 3 provides a concise overview of the estimated parameters for the DDM achieved through the Ls-AOA. The results in Table 3 illustrate the Ls-AOA’s proficiency in parameter estimation, demonstrating its capability to attain a high degree of accuracy. Notably, the RMSE value obtained is low, emphasizing the Ls-AOA’s ability to precisely estimate the I-V characteristics of the DDM model, ultimately leading to improved model performance. Additionally, Figs 9 and 10 visually depict the I-V and P-V curve characteristics of the DDM optimized using the Ls-AOA. These graphical representations illustrate the effectiveness of the optimized model, as evidenced by the close alignment of the curves with the experimental data.

**Table 3:** Estimated parameters of DDM with Ls-AOA.

*I*_*ph*_ (A)	*I*_*sd*1_ (μA)	*I*_*sd*2_ (μA)	*R*_*s*_ (Ω)	*R*_*sh*_ (Ω)	*n*_1_	*n*_2_	RMSE
0.7608	0.1007	1.0000	0.0375	55.8959	1.3890	1.8409	7.4241E−04

### Simulation results of TDM

**Fig 11:**
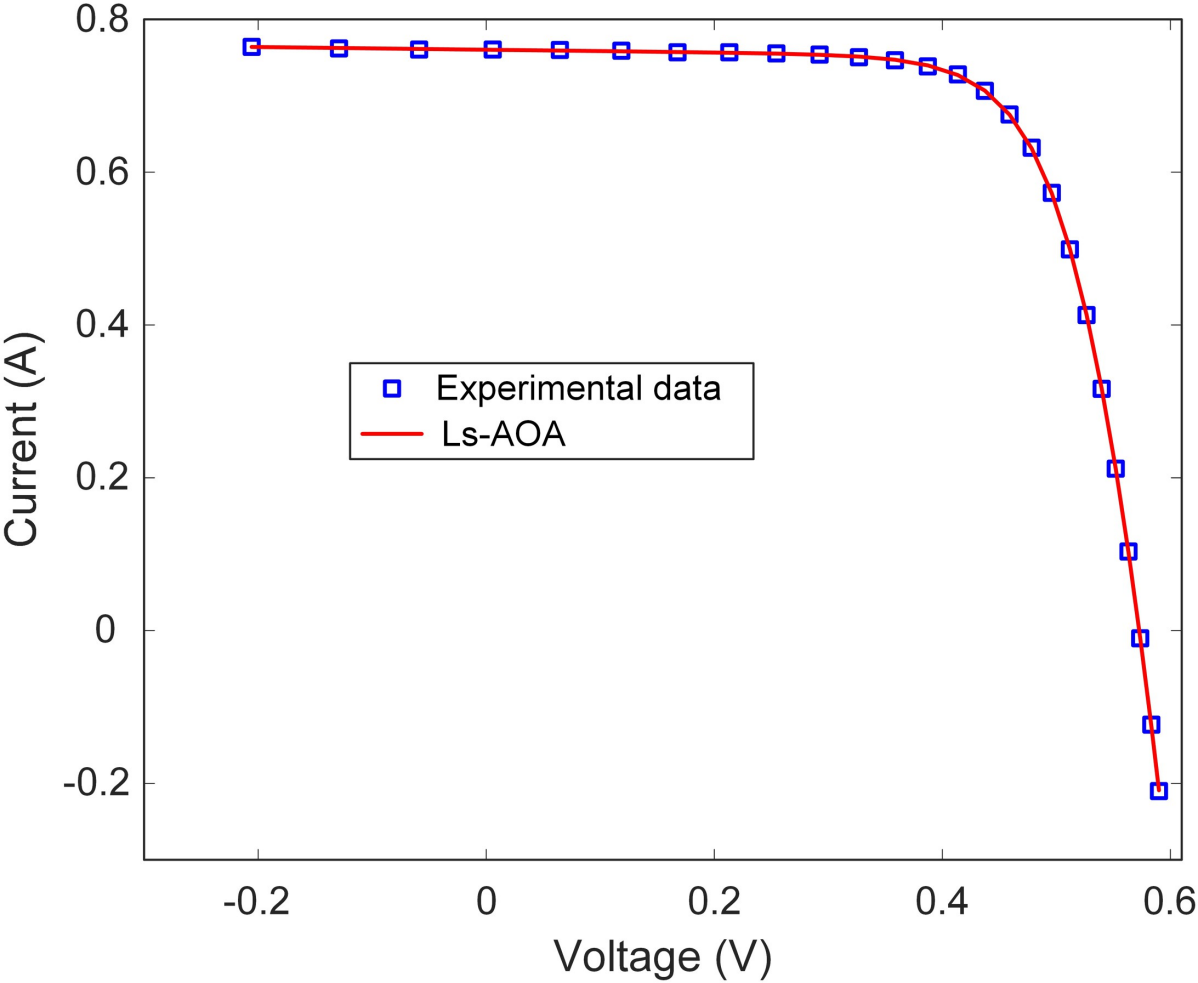
I-V curve characteristics of TDM.

**Fig 12:**
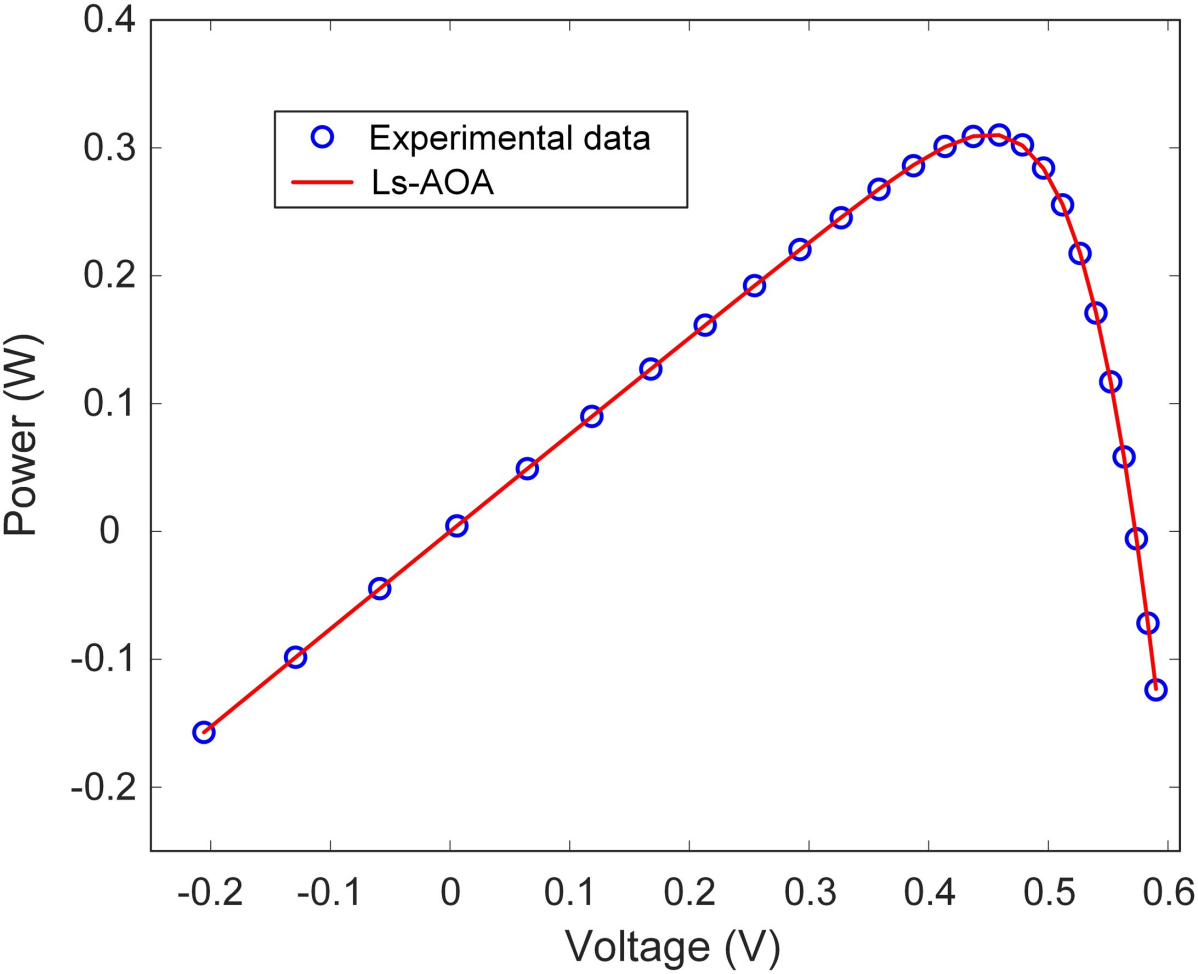
P-V curve characteristics of TDM.

This section presents the simulation results for the TDM optimization using the Ls-AOA. Table 4 succinctly presents the estimated parameters for the TDM model as determined through the Ls-AOA approach. The results presented in Table 4 demonstrate the Ls-AOA’s effectiveness in parameter estimation, with a notable degree of precision achieved. We include the RMSE value to further gauge the accuracy of the TDM optimization. Crucially, the obtained RMSE value is low, highlighting the Ls-AOA’s ability to accurately estimate the I-V characteristics of the TDM model, ultimately resulting in improved model performance. Moreover, Figs 11 and 12 graphically illustrate the I-V and P-V curve characteristics of the TDM optimized using the Ls-AOA. These visual representations exemplify the effectiveness of the optimized model, as they closely align with the experimental data, affirming the precision and suitability of the Ls-AOA for parameter optimization in the TDM context.

**Table 4:** Estimated parameters of TDM with Ls-AOA.

*I*_*ph*_ (A)	*I*_*sd*1_ (μA)	*I*_*sd*2_ (μA)	*I*_*sd*3_ (μA)	*R*_*s*_ (Ω)	*R*_*sh*_ (Ω)	*n*_1_	*n*_2_	*n*_3_	RMSE
0.7608	0.1028	0.4894	0.7993	0.0377	56.4046	1.3888	1.9658	1.8722	7.3882E−04

### Statistical performance evaluation of Ls-AOA

In this section, a comprehensive statistical evaluation of the Ls-AOA’s performance across three distinct photovoltaic models (SDM, DDM, and TDM) is conducted. Table 5 provides a detailed summary of the statistical performance metrics for these models. The table summarizes four key statistical performance metrics (average, standard deviation, best. and worst) for each model. These statistics provide a comprehensive view of the Ls-AOA’s performance across different PV models. The low average RMSE values, coupled with small standard deviations, signify consistent and accurate parameter estimation. Additionally, the best and worst RMSE values showcase the algorithm’s ability to perform well even under challenging conditions.

**Table 5:** Statistical performance of Ls-AOA for SDM, DDM and TDM.

Model	Average {RMSE}	Standard deviation {RMSE}	Best {RMSE}	Worst {RMSE}
SDM	7.7510E−04	4.3640E−06	7.7299E−04	7.8942E−04
DDM	7.6459E−04	1.0531E−05	7.4241E−04	7.7790E−04
TDM	7.4806E−04	6.9498E−06	7.3882E−04	7.6596E−04

### Convergence performance evaluation of Ls-AOA

**Fig 13:**
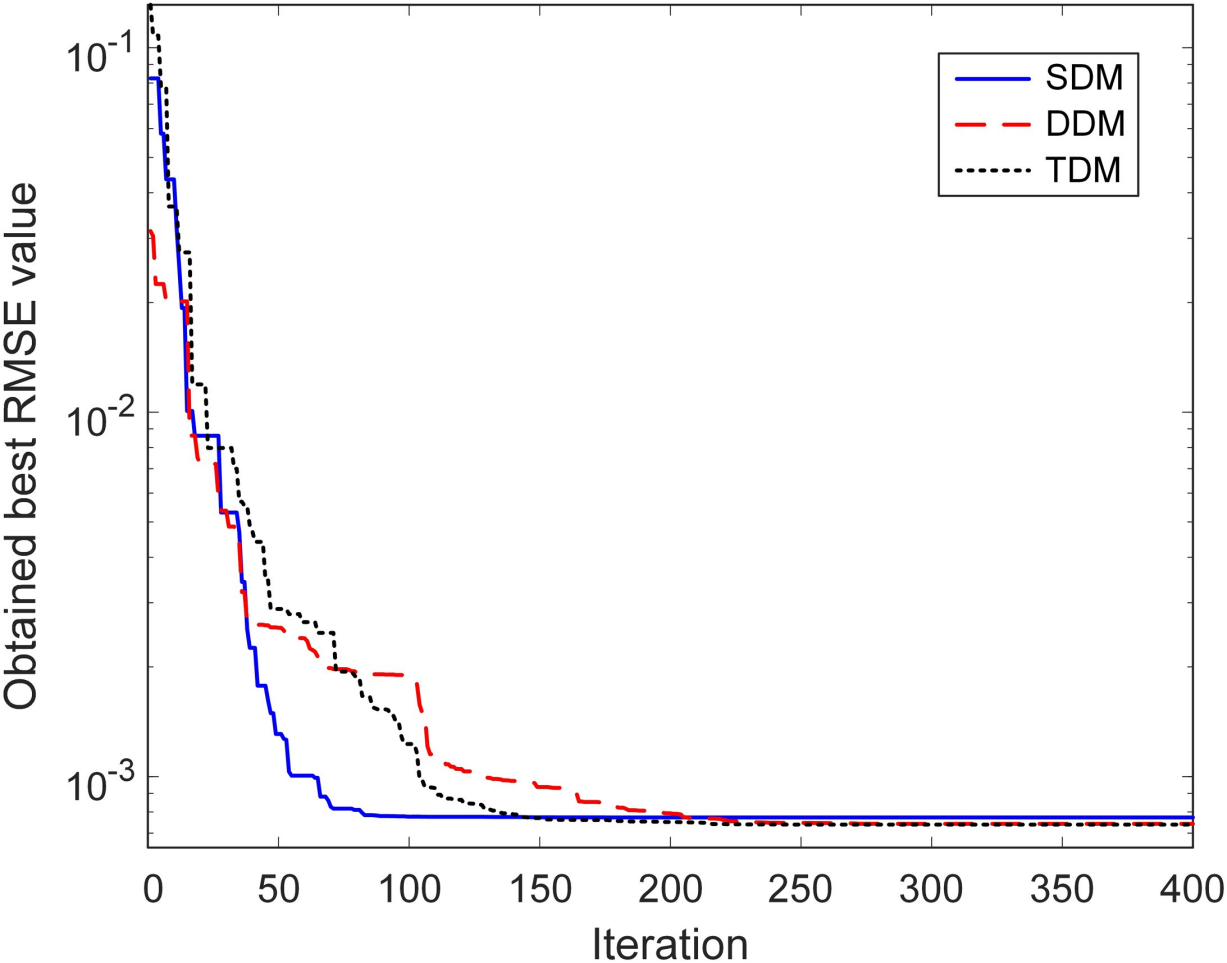
Convergence behavior of Ls-AOA.

In this section, the evaluation of the convergence performance of Ls-AOA across the SDM, DDM, and TDM for RMSE optimization is presented. One significant aspect of assessing algorithm performance is to scrutinize its convergence behavior. Ls-AOA exhibits a remarkable capability to converge toward the lowest obtained RMSE values across SDM, DDM, and TDM. As depicted in Fig 13, we observe the convergence behavior of Ls-AOA.

It demonstrates swift convergence towards the lowest RMSE value for the SDM, achieving this milestone in earlier iterations compared to both the DDM and TDM. This rapid convergence underscores Ls-AOA’s efficiency in fine-tuning the SDM parameters. Furthermore, the figure illustrates that Ls-AOA converges to the lowest RMSE value for the TDM earlier than it does for the DDM. This observation highlights the algorithm’s ability to adapt its convergence pattern according to the intricacies of different models, optimizing them effectively and efficiently.

### Computational complexity of Ls-AOA

In this section, the computational complexity of the proposed Ls-AOA is discussed. The computational complexity of the original AOA can be described as *O*(*AOA*) = *O*(*N*) + *O*(*N* × *t*_*max*_) + *O*(*N* × *Dim* × *t*_*max*_) where *N* represents the population size, *t*_*max*_ denotes the maximum iteration number and *Dim* signifies the number of variables in the optimization problem. The proposed Ls-AOA, however, incorporates logarithmic spiral search and greedy selection mechanisms additionally compared to the original AOA. Consequently, the computational complexity in this case is provided as *O*(*Ls–AOA*) = *O*(*AOA*) + *O*(*N* × *t*_*max*_).

The comparative computation times of AOA and Ls-AOA for SDM, DDM, and TDM solar PV models are listed in Table 6. Upon examination of the numerical values in the table, it is evident that the average computation times for the Ls-AOA show an increase between 2–3% compared to the AOA which can be considered as negligible. The slight increase of the computational times for Ls-AOA is expected due to additional mechanisms incorporated within the original AOA, however, due to integration approach, it is not significant. Besides, considering the significant performance improvement, this slight increase can be ignored.

**Table 6:** The average computation times of AOA and Ls-AOA for each run.

Model	AOA	Ls-AOA
SDM	14.6948 s	14.9958 s
DDM	14.9450 s	15.3295 s
TDM	15.2399 s	15.5936 s

### Comparison with other metaheuristic algorithms

In this section, the performance of the Ls-AOA algorithm with a range of other established metaheuristic algorithms in the context of optimizing SDM, DDM, and TDM solar PV models is compared. Table 7 presents the numerical performance analysis of Ls-AOA with respect to other metaheuristic-based approaches. The evaluated algorithms include hybrid multi-group stochastic cooperative particle swarm optimization (HMSCPSO) [28], improved learning search algorithm (ILSA) [29], generalized oppositional teaching learning based optimization (GOTLBO) [30], teaching—learning—based artificial bee colony (TLABC) [31], inspired grey wolf optimizer (IGWO) [32], improved opposition-based whale optimization algorithm (OBWOA) [33], sunflower optimization algorithm (SFO) [34], gradient-based optimizer (GBO) [35], spherical evolution (SE) [36], slime mould algorithm (SMA) [37], atom search optimization (ASO) [38], comprehensive learning particle swarm optimizer (CLPSO) [39], particle swarm optimization (PSO) [40], hybrid rat swarm optimization and pattern search (hARS-PS) [41], modified salp swarm optimization (MSSA) [42], hybrid single candidate optimizer and chaotic sand cat optimizer (CSCSC) [43] and improved tunicate swarm optimization (ITSA) [44].

**Table 7:** Numerical performance analysis of Ls-AOA with respect to other metaheuristic based approaches.

Model	Algorithm	Average {RMSE}	Standard deviation {RMSE}	Best {RMSE}	Worst {RMSE}
SDM	Ls-AOA	7.7510E−04	4.3640E−06	7.7299E−04	7.8942E−04
	HMSCPSO	9.8602E−04	5.7282E−15	9.8602E−04	9.8602E−04
	ILSA	1.3851E−03	6.8799E−04	9.9977E−04	4.4647E−03
	GOTLBO	9.8602E−04	7.8549E−12	9.8602E−04	9.8602E−04
	TLABC	9.9642E−04	1.2797E−05	9.8608E−04	1.0452E−03
	IGWO	6.6947E−03	7.4765E−03	1.6799E−03	4.1120E−02
	OBWOA	1.9502E−03	1.1035E−03	9.8960E−04	7.0588E−03
	SFO	3.1403E−03	2.0117E−03	1.0027E−03	9.5160E−03
	GBO	9.8602E−04	1.0895E−12	9.8602E−04	9.8602E−04
	SE	2.4396E−03	1.5243E−06	2.4378E−03	2.4430E−03
	SMA	1.9438E−03	5.5692E−04	1.2064E−03	3.8140E−03
	ASO	3.0480E−03	3.2805E−03	1.2608E−03	1.4181E−02
	CLPSO	1.0047E−03	3.6466E−05	9.8615E−04	1.1402E−03
	PSO	1.8551E−02	3.9068E−02	1.4385E−03	2.0699E−01
	hARS‐PS	9.8500E−04	3.0100E−07	9.8400E−04	9.8700E−04
	MSSA	9.8600E−04	3.0100E−07	9.8500E−04	9.8700E−04
	CSCSC	9.8602E−04	1.1231E−09	9.8602E−04	9.8602E−04
	ITSA	9.8900E−04	3.0100E−07	9.8600E−04	8.0700E−03
DDM	Ls-AOA	7.6459E−04	1.0531E−05	7.4241E−04	7.7790E−04
	HMSCPSO	9.8521E−04	1.2717E−06	9.8249E−04	9.8768E−04
	ILSA	2.0371E−03	1.2283E−03	1.0051E−03	6.4631E−03
	GOTLBO	9.8471E−04	1.2777E−06	9.8262E−04	9.8691E−04
	TLABC	1.0897E−03	2.8773E−04	9.8644E−04	2.4480E−03
	IGWO	6.0593E−03	7.3472E−03	1.9197E−03	4.2447E−02
	OBWOA	2.0414E−03	6.5149E−04	1.1088E−03	4.0192E−03
	SFO	5.3534E−03	6.2657E−03	1.1743E−03	2.5992E−02
	GBO	1.0093E−03	8.9548E−05	9.8250E−04	1.3397E−03
	SE	1.9864E−03	4.3562E−04	9.9891E−04	2.4435E−03
	SMA	2.0125E−03	6.1928E−04	1.0477E−03	4.5841E−03
	ASO	3.3761E−03	2.3006E−03	9.9236E−04	1.1281E−02
	CLPSO	1.0631E−03	1.5193E−04	9.8608E−04	1.7587E−03
	PSO	1.4217E−02	1.7652E−02	9.8286E−04	4.3495E−02
	hARS‐PS	9.8400E−04	1.4500E−06	9.8200E−04	9.8700E−04
	MSSA	9.9400E−04	1.4900E−06	9.8300E−04	9.9900E−04
	CSCSC	9.862E−04	1.2801E−06	9.8350E−04	9.877E−04
	ITSA	9.8200E−04	1.4500E−06	9.8000E−04	8.3400E−03
TDM	Ls-AOA	7.4806E−04	6.9498E−06	7.3882E−04	7.6596E−04
	HMSCPSO	9.8449E−04	1.7012E−06	9.8249E−04	9.8875E−04
	ILSA	3.7149E−03	5.1369E−03	1.0906E−03	2.9440E−02
	GOTLBO	9.8513E−04	2.0857E−06	9.8290E−04	9.9154E−04
	TLABC	1.1365E−03	2.9090E−04	9.8916E−04	2.3912E−03
	IGWO	6.9433E−03	1.0136E−02	1.8593E−03	5.8416E−02
	OBWOA	2.4524E−03	8.0763E−04	1.0370E−03	3.7377E−03
	SFO	3.7184E−03	2.0384E−03	1.0480E−03	9.3214E−03
	GBO	1.0101E−03	6.5732E−05	9.8252E−04	1.3008E−03
	SE	1.7261E−03	4.7103E−04	1.0384E−03	2.5241E−03
	SMA	2.0641E−03	7.3098E−04	9.8790E−04	4.7632E−03
	ASO	3.8224E−03	2.7252E−03	1.5422E−03	1.4815E−02
	CLPSO	1.0626E−03	1.3945E−04	9.8718E−04	1.6584E−03
	PSO	3.1565E−02	4.9226E−02	9.8867E−04	2.2286E−01

For the SDM, Ls-AOA achieves an average RMSE of 7.7510E−04 with a low standard deviation of 4.3640E−06. It obtains the best RMSE value of 7.7299E−04, which is highly competitive among all algorithms, and a worst-case RMSE of 7.8942E−04. Comparatively, other algorithms exhibit higher average RMSE values, indicating less accurate parameter estimation. For instance, ILSA reports an average RMSE of 1.3851E−03, significantly higher than Ls-AOA’s result. Ls-AOA also demonstrates strong performance in DDM optimization, with an average RMSE of 7.6459E−04 and a small standard deviation of 1.0531E−05. Its best RMSE is 7.4241E−04, showcasing its robustness in parameter estimation. In contrast, several other algorithms produce higher average RMSE values for DDM, indicating less accuracy in capturing the behavior of the solar cell. For the TDM, Ls-AOA maintains its accuracy, reporting an average RMSE of 7.4806E−04 with a standard deviation of 6.9498E−06. It achieves the best RMSE of 7.3882E−04, demonstrating its consistency in accurately estimating parameters. Most other algorithms yield higher average RMSE values for the TDM model, suggesting that Ls-AOA excels in optimizing this model. The comparison across the three models underscores the superiority of Ls-AOA in terms of achieving lower RMSE values, indicating better accuracy in parameter estimation compared to other metaheuristic algorithms. These results validate Ls-AOA as a highly competitive and efficient approach for optimizing solar PV models, with the potential to significantly contribute to the field of solar energy research and development.

## Conclusion

This work has focused on the crucial problem of estimating parameters in photovoltaic (PV) models, aiming to accurately predict solar energy systems. The accurate characterization of PV systems is crucial for effectively capturing solar energy. This relies on estimating the hidden parameters inside these models. This work presents the Ls-AOA as a novel and powerful metaheuristic technique for parameter estimation in PV models. Building upon the foundation of the AOA, Ls-AOA incorporates logarithmic search behavior and a selective mechanism. This combination improves the algorithm’s ability to explore, making it a powerful tool for obtaining accurate parameters in PV models. The study centered its investigation on the RTC France solar cell as a benchmark case study. By implementing a consistent experimental framework, the Ls-AOA method was smoothly included into the parameter tuning process for three separate PV models: SDM, DDM, and TDM. The thorough examination and interpretation of data required not only statistical analysis but also assessments of convergence behavior. The results indicated that Ls-AOA consistently produced low RMSE values, indicating its better performance in properly predicting the I-V properties of the examined PV models. The efficient performance of Ls-AOA is further confirmed by its smooth convergence behavior. Comparative analyses with alternative methods, including HMSCPSO [28], ILSA [29], GOTLBO [30], TLABC [31], IGWO [32], OBWOA [33], SFO [34], GBO [35], SE [36], SMA [37], ASO [38], CLPSO [39], PSO [40], hARS-PS [41], MSSA [42], CSCSC [43] and ITSA [44], unequivocally established Ls-AOA’s competitive edge in the realm of solar PV model parameter optimization.
